# Supplementary material for: Development & validation of a health awareness booklet: “Reproductive health schemes for tribal women of Jharkhand” a study protocol
Source: PLoS One. 2025 May 5;20(5):e0322777. doi: 10.1371/journal.pone.0322777 (PMC12052126; doi:10.1371/journal.pone.0322777)
Supplement: S1 File — (DOCX) [file pone.0322777.s001.docx]

**Protocol (Description of the Project)**

**1. Title of the project:** Development & validation of a health awareness booklet: “Reproductive Health Schemes for tribal women of Jharkhand”

**2. Type of Study:**  Sequential Explanatory Design (1)

3. **Departments involved**: Community Medicine

4.**Proposed total duration of the proposal:** 36 months

5.**If animals involved-** NA

## 6. Aims & objectives:

Aim: To study the utilization of healthcare schemes among tribal reproductive age group population in Jharkhand.

## Objectives:

**Phase 1: Baseline Assessment**

- To assess the knowledge, attitude, and practices of healthcare schemes among tribal women in the reproductive age group.
- To understand the challenges faced in provision of tribal reproductive age group women healthcare schemes from different Stakeholders perspective.

**Phase 2: Intervention**

- To develop & validate the awareness booklet for the tribal women of reproductive age group and evaluate the perception of participants towards it.

## Give a time line for each of your aims/ in tabular format.

| **Research Activity** | **Time in Months (from admission)** | | | | | | | | |
| --- | --- | --- | --- | --- | --- | --- | --- | --- | --- |
|  | 1-6 | 6-12 | 12-18 | | 18-24 | 24-30 | | 30-36 | 36-42 |
| **Course Work** |  |  |  | |  |  | |  |  |
| **Literature Survey** |  |  |  | |  |  | |  |  |
| **Methodology Step 1** |  |  |  |  |  |  | |  |  |
| **Methodology Step 2** |  |  |  |  |  |  |  |  |  |
| **Methodology Step 3** |  |  |  | |  |  |  |  |  |
| **Methodology Step 4** |  |  |  | |  |  | |  |  |
| **Half Yearly Reports** |  |  |  | |  |  | |  |  |
| **Conference presentations** |  | **C** |  | |  |  | | **C** |  |
| **Journal Publications** |  |  | **J** | |  |  | | **J** |  |
| **Preparation of Final Thesis** |  |  |  | |  |  | |  |  |
| **Synopsis presentation** |  |  |  | |  |  | |  |  |
| **Submission of Thesis** |  |  |  | |  |  | |  |  |

## 7. Justification for study (whether of national significance with rationale):

1**. Disparity in Maternal Health care utilization:**

Maternal mortality Ratio of Jharkhand state showed a declining trend (56 per lakh live births) compared to India (97 per lakh live births) as per SRS: 2018-20.(2) However utilization of Maternal Child Health services in Jharkhand state is lagging behind. National Health Family Survey 5 data showed the percentage of mothers who had at least 4 antenatal care visits is 54.2 in India, whereas it is only 36.4% in Jharkhand. Similarly rural Jharkhand have 92.3% of registered pregnancies, but only 36.4 % had 4 ANC visits, 13.2% of mothers consumed iron folic acid for 180 days or more when they were pregnant, and 66.7% of mothers received postnatal care.

2. **Lack of accessibility to safe menstrual health**

A study context to rural part of West Singhbhum found that 38 % of the adolescent girls found to be anemic, and 27% of the adolescent girls uses local napkins during their menstruation.^(3)^

**3. Malnutrition and reproductive health**

Anaemia in pregnant women (15-49 years old) is 59.2% and in adolescent girls (15-19 years) is 66.7% respectively according to Jharkhand NFHS 5 report, this is slightly higher from Indian context with 54.3% and 60.2% respectively. Whereas it was found that rural women with Body Mass Index below normal was 29.2% as compare to overall India context with 21.2%.

**4**. **Uncontrolled fertility**

According to NFHS 5 report, the percentage of male sterilization is 0.2% in rural areas of Jharkhand followed by female sterilization 0.4%, condom usage 3.5%, and pills usage 3.1% only. Health workers talked to female non users about family planning in rural area of Jharkhand is 29.5%.

Therefore, Research on the knowledge, awareness and attitudes towards health coverage schemes in tribal regions of India is essential for addressing health disparities. Improving awareness and understanding, overcoming attitudinal barriers, informing policy development, and promoting equity and social justice in healthcare are some of the disparities. It can contribute to designing effective interventions and policies that cater to the specific needs of tribal communities, ultimately improving their access to quality healthcare services. In Jharkhand the public health facilities utilization is still less than the national average.^(4)^ Hence, this study will help to increase in knowledge, awareness, and attitude for reproductive age group tribal women health coverage schemes in the regional language with more pictorial content that can be understood easily.

8. Is this a new study or does it build on previous work by investigators? New study

**9. Departments involved:** Community Medicine

List all the names and designations of the investigators, with official email addresses and phone numbers, and signatures (e-signatures are permitted if followed up with email to Research Cell):

| S.No. | Name | Designation | E mail |
| --- | --- | --- | --- |
| 1. | Dr. Rohit Raj (PI) | Ph.D. Scholar | [rohit1.mtmcjsr2022@learner.manipal.edu](mailto:rohit1.mtmcjsr2022@learner.manipal.edu) |
| 2. | Dr. Jarina Begum (Co-PI) | Professor & Head | [jarina.begum@manipal.edu](mailto:jarina.begum@manipal.edu) |

10. **First Authorship**: Dr. Rohit Raj

11. **Corresponding author**: Prof. Dr. Jarina Begum

12. If any **new investigator** is added in the project after approval by Institutional scientific committee (ISC)/ IEC- resubmit the proposal with the additional details clearly mentioned: NA

13. **Division of labour**:

| **S.No.** | **Work to be done** | **Name of the Investigator** |
| --- | --- | --- |
| 1. | Idea of Study | Dr.Jarina Begum, Dr.Rohit Raj |
| 2. | Design of study | Dr. Rohit Raj, Dr. Jarina Begum |
| 3. | Proposal writing | Dr.Rohit Raj, Dr. Jarina Begum |
| 4. | Review of Proposal | Dr.Jarina Begum |
| 5. | Questionnaire selection & designing | Dr.Rohit Raj, Dr.Jarina Begum |
| 6. | Data Collection | Dr.Rohit Raj |
| 7. | Data Compilation & Analysis | Dr.Rohit Raj |
| 8. | Report Writing | Dr.Rohit Raj |
| 9. | Proof reading of report | Dr.Jarina Begum |

14. **Proposed publication**:

a) Plos One

b) Globalization and Health

c) Journal of Health Services Research and Policy

d) Health Behavior and Policy Review.

15. **Time line for publication:**3 months

16. **External funding accepted**: NA

17. **Plan for submission for external funding**: NA

18. **Presentation at conference:** Yes

Manipal Colliquium, year 2025

Who is the likely presenting author: Dr. Rohit Raj

19. **Patents:** If patent is likely after completion of the project, consent to resubmitting the proposal after contacting Research office for additional paperwork to be filled out: Yes (patent of Health awareness booklet)

20. **Preliminary data/ experience of the investigators** in being able to conduct the research: NA

21. **Interinstitutional document of consent** to be signed by all investigators and Heads of the institutes: NA

22. **Ongoing submission of work in progress:** Please give your consent for 6 monthly submissions of data generated to be submitted to the ISC if the proposal is more than a year old: Yes

23. Delays/ extensions requested: Please give your consent that in case of any delays, justification will be given in case of extension of deadline, signed by all investigators: Yes

24. Attach any **questionnaire** if relevant to the study: Annexure 1, Draft questionnaire

**25. Study period:** 36 Months

## 26. Sample size: Give the statistical explanation and formula used to arrive at the sample size.

​

Formula used: n = (Z_1-α/2_)^2^ (p) (1-p)/d^2^ = 385

Sample size-740: The sample size was calculated through an online tool *“calculator.net”* keeping the confidence interval(z) at 95%, and marginal error(E)at 5% and population size 258454**.**^(5)^ Considering the drop-out of 20% and design effect 1.5 the sample size becomes 722 rounding up to 740.

Sampling method – Simple random Sampling and stratified sampling (Flow chart mentioned below in figure1.)


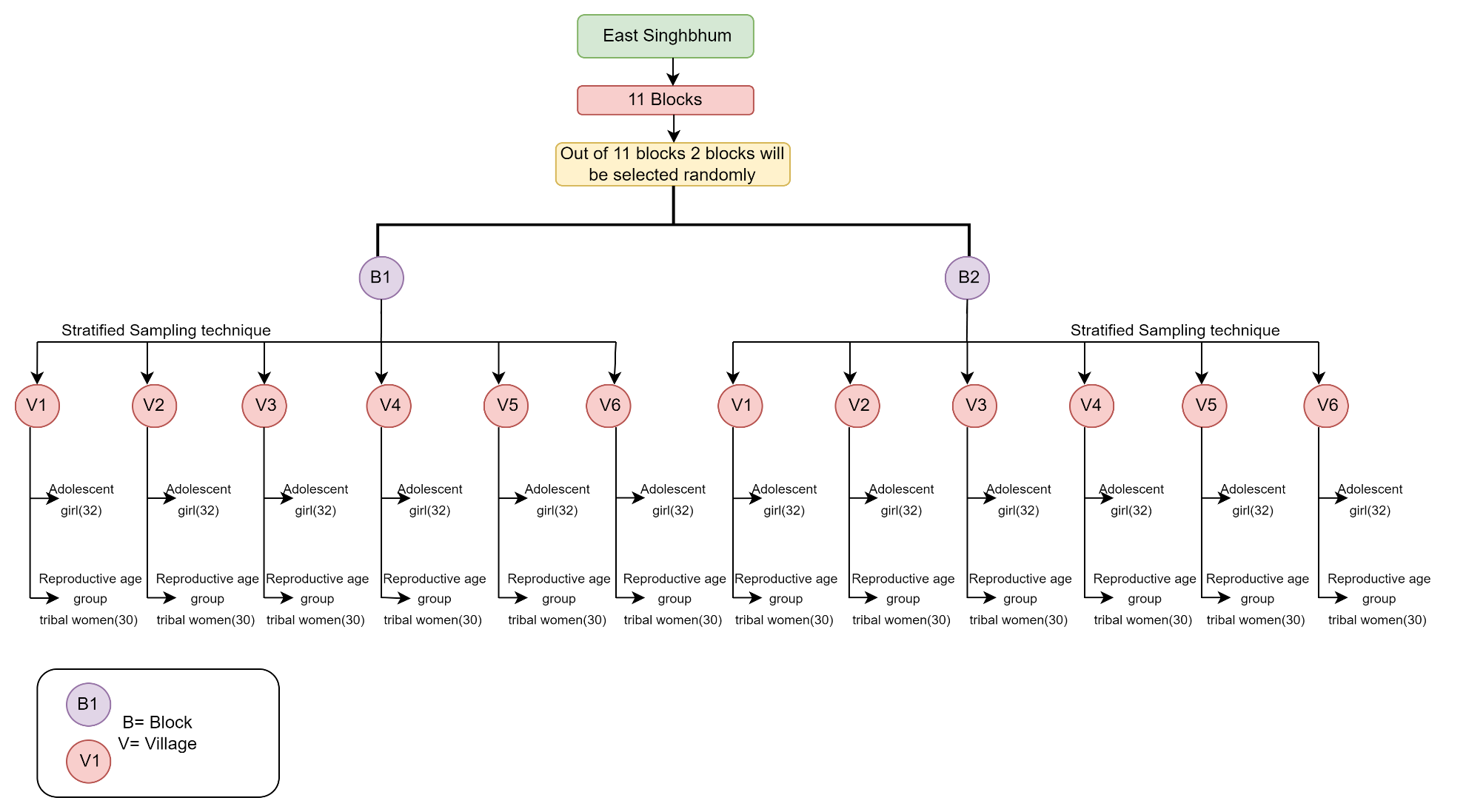


Figure 1: Representation flow chart of the sampling method

For qualitative data – purposive sampling will be done. (8 interviews per each village for various stake holders)

**27. Materials and methods:**

a) Inclusion and exclusion criteria:

**Inclusion criteria:**

1. Adolescent girls (10-19years) & tribal women in the reproductive age group (15 to 49years), belonging to the following tribes: Santhal, Ho, Munda, Bhumiz, Kharia, and Sabar.

2. Who gave consent to participate in the study.

**Exclusion criteria:**

1. Migrant population and tribal women suffering from any mental illness.

b) Biological materials required: NA

c) Statistical methods:

For Objective 1: Quantitative data analysis in terms of frequency, percentage, proportion and for categorical data chi square test

Objective 2: qualitative study, analysis based on themes and codes.

Objective 3: Face and content validity tools, Descriptive analysis after the training sessions to evaluate the pre-post test and feedback from participants.

d) Tools used:

Objective 1: Semi-structured pre-validated questionnaire, analysis through Jamovi software (Version solid 2.2.5).

Objective 2: Semi-structured validated, open ended questions for in-depth interviews

Analysis through MaxQda software.

Objective 3: Develop and validate booklet, validation through experts, and by piloting. Feedback questionnaire form analysis through Jamovi software.

**28. Detailed description of procedure/processes:**

- Study will start after IEC and university registration.
- Phase 1 will have base line survey will be done to assess the knowledge, attitude, and practices of healthcare schemes among tribal adolescent girls, and reproductive age group women.
- In-depth interview will be conducted to understand the challenges faced in provision of adolescent and reproductive age group women healthcare schemes from different Stakeholders perspective such as block program manager/officer, block trainer, *Accredited Social Health Activist* (ASHA), *Auxiliary Nurse Midwifery* (ANM), *Aanganwadi Workers* (AWW), Medical Officer, Child Development Project Officer, reproductive age group women, and Gram Mukhiya. 5-8 interviews per each village will be done till the saturation of data over a period of 1 year to collect and analyze the data further.
- In Phase 2 based on the results of survey among tribal women and in-depth interview of all stake holders, an tri linguistic (English, Hindi, and Santali) language awareness booklet will be developed on eight health schemes targeted for reproductive age group tribal women. It will be validated by the subject experts and piloted inform of training sessions & through A/V clips, among the study participants.
- Pre-test and post-test will be done to know the improvement knowledge based on the booklet.
- This will be followed by evaluation of the feedback of participants towards the awareness booklet through feedback form.

**29. Outcome measures:**

- Increase in knowledge, attitude and practice towards various health schemes (8) among tribal women.
- Development of Booklet covering the health schemes targeted for tribal women.
- Booklet can be copyrighted and can be used by others in larger study population.
- This research would provide valuable insights for policymakers, healthcare providers, and implementers to tailor the programs to the specific needs and preferences of the tribal communities, ensuring their effective utilization and impact.

**Outcome Indicators:**

| **Outcomes** | **Indicators** | **Data collection method** |
| --- | --- | --- |
| Baseline data (KAP) of health care schemes utilization among tribal women of reproductive age group | % of Tribal women aware of the (8) health schemes.  % of Tribal women felt the 8 health schemes beneficial  % of Tribal women felt the importance of cash incentive provided through the health schemes  % of Tribal women rated their experience as excellent. | KAP Survey Questionnaire |
| Identifying challenges in utilization of services from different stake holders | Challenges identified at various levels of Operations, Utilizations, Process management, Enablers, Maintenance, & Sustainability | In-depth Interview |
| Development & validation of Booklet covering the health schemes targeted for tribal women | Booklet developed in tri linguistic language.  Booklet validated by external experts. | Digital designing Tool & language experts  Face & Content validity |
| Increase in knowledge among tribal women after the training program on various health schemes (8) mentioned in the booklet. | Increase in the average knowledge regarding health schemes (8) among the tribal women. | Pre-post test Questionnaire |
| Participants satisfied with the process of training.  Participants perceived the content of booklet effective. | % of participants satisfied with the process of training.  % of participants perceived that the content of booklet is effective. | Feedback survey |
| Booklet can be copyrighted and used by policy makers for implementation at larger level for tribal communities ensuring effective utilization of the health schemes. | Booklet advised to be adopted and implemented at district and state level by Govt. authorities and MAHE University. | Submission to state: Ministry of Health and Family Welfare, Department of Schedule Tribe, Schedule Caste, Minority, and Backward Class Welfare, MAHE University |

## 30. Potential risks and benefits: Minimal risk

## Potential risks:

- Lack of cooperation from tribal population.
- Language barrier may in-counter in tribal region.

Risk mitigation strategies:

- On-site field workers can be approached to reach the population.
- Pictorial presentations could be incorporated for better understanding.

Potential benefits:

Community at large may be benefitted by the intervention and recommendations of this study based on the findings.

31. What are the limitations of your study? How would you address these, while still keeping in line with your original goals?

Generalizability of the findings may be a limitation as this study will be done in small sample of population due to constraints of resources, but this study can act as a baseline study based on which some larger studies may be planned to get more generalizable findings.

## 32. Ethical considerations and methods to address issues: Permission of IEC will be taken from institute ethics committee.

33. Please provide consent form for your study: Under process

**34. Budget (give details) and proposed funding source:** Nil

**35. Review of literature (within 1000 words):**

Women's reproductive health is a significant issue in India. To assure the population's growth and development, the country has lot of work to perform. Many studies on women's health coverage programs in India have been done to understand the problems. Over the past 20 years, India has experienced increased economic growth, but it has performed poorly in terms of health and human development indexes. Access to healthcare and significant health inequities have become worse and persisted across states' communities. There are some major health coverage schemes running in India to improve the health of a reproductive age group women, such as *Janani Suraksha Yojana, Janani Sishu Swasthya Karyakram, Pradhan Mantri Matri Vandana Yojana, Rashtriya Kishori Swasthya Karyakram, Poshan Abhiyan, Anaemia Mukt Bharat.*

According to a survey study conducted in India by Bango et al.^(6)^, the study discovered considerable caste/tribe disparities in the use of Maternal Child Health care services in the chosen states of *India*. The main causes of the under-utilization of these services, particularly for members of disadvantaged social groups, were limited accessibility and the absence of comprehensive healthcare. The outcome also indicated that it would be dangerous to confirm "*Health for All*" right away. It will be the effectiveness with which India addresses healthcare service disparities and ensures high-quality care.^(6)^

Whereas Ali et al.^(7)^ did a survey study to analyze the level of disparity in maternal health care, including complete Antenatal Care (full ANC), Skilled Attendants at Birth (SBA), and Postnatal Care (PNC) in rural India. The study found that maternal health care utilization in rural India decreased significantly across socioeconomic levels. The usage of maternal health care, particularly the use of SBA, was found to have significantly improved. The concentration index for SBA significantly decreased over the course of this decade, whereas the complete ANC concentration index decreased over the same time period, falling from 0.47 to 0.32, and the full ANC showed the least reduction in inequality. Decomposition analysis's findings also revealed that scheduled tribes, media exposure, secondary and higher education, and secondary and higher education contributed significantly to the inequality.^(7)^

A qualitative study was done by Pannu et al.^(8)^ in Uttar Pradesh regarding “*Accessibility of Reproductive Health Related Schemes for Pregnant and Lactating Rural Women*”, it was found that the study focused into the way women experienced access and hurdles in using health care schemes. Networks linking women to the schemes were part of it. In this study, assumptions and qualitative research categories are addressed. It describes the biases that develop when 'rural' women's reproductive health is being studied. Gender inequality is exacerbated by poverty's vicious cycle. It keeps having a negative impact on human flourishing and eroding human potential. Economic changes typically occur simultaneously with cultural and institutional changes over a protracted period of time. The government must continue to put a serious effort into enhancing service delivery until gender inequities in access to vital healthcare during the reproductive phase are overcome.^(8)^

A study done by Chauhan et al.^(9)^ in the three Indian states of Madhya Pradesh, Jharkhand, and Chhattisgarh, which are inhabited by tribes. The goal of the current study was to investigate the variables influencing the use of maternal health services. The National Family Health Survey (NFHS-4) data were used in the study. According to the study's findings, there were wider socio-economic disparities in the use of MCH services (full ANC, Safe delivery, and post-natal care) in each of the three states that were the subject of the investigation. According to the regression analysis, tribal people are less likely than other Caste groups to use maternity and child health care facilities.^(9)^

In a study done by Thongkong et al.^(10)^ measure and explain socioeconomic inequality in the receipt of JSY benefits from Jharkhand and Odisha. While the majority of women (94% in Odisha and 85% in Jharkhand) were aware of the programs, only 62% in Odisha and 20% in Jharkhand were receiving benefits from it. The percentage of women who obtained the benefits varied greatly by district, particularly in Jharkhand, where 5% of women in Godda district and 40% of women in Ranchi district did so. Delivery at a public facility was a significant factor in determining the receipt of JSY benefits and contributed significantly to the explanation of the observed disparities between the rich and the poor in benefit receipt. On the other hand, in Odisha, poorer women giving birth in a government facility had a same chance of receiving JSY benefits as affluent women.^(10)^

Another study was done by Chakraborty S.^(11)^ states that one of India's most fragile and impoverished states is Jharkhand. There aren't many community-based programs and campaigns that prioritize adolescent and youth public health issues like gender and sexuality. This essay draws on observations made over three years of engagement with adolescents and young people in Jharkhand's rural communities on issues related to gender and sexual and reproductive health and rights (SRHR). A technology-based health programs on gender, sex, and sexuality in various Jharkhand villages is covered in the first section of the article. The second section of the article discusses accounts and the advocacy project Periods pe Charcha (Let's speak Periods), a free online and offline campaign to make menstrual hygiene services for young girls mandatory. This essay argues for the necessity for more organised efforts and campaigns on SRHR for young people at grassroots levels in the state while also highlighting some shortcomings in current policies and programmes.^(11)^

**36. References:**

1. Ivankova N V., Creswell JW, Stick SL. Using Mixed-Methods Sequential Explanatory Design: From Theory to Practice. http://dx.doi.org/101177/1525822X05282260. 2006 Feb 1 [cited 2023 Aug 9];18(1):3–20. Available from: https://journals.sagepub.com/doi/10.1177/1525822x05282260

2. India - SAMPLE REGISTRATION SYSTEM (SRS)-SPECIAL BULLETIN ON MATERNAL MORTALITY IN INDIA 2018-20. [cited 2023 Aug 9]. Available from: https://censusindia.gov.in/nada/index.php/catalog/44379

3. Rose-Clarke K, Pradhan H, Rath S, Rath S, Samal S, Gagrai S, et al. Adolescent girls’ health, nutrition and wellbeing in rural eastern India: a descriptive, cross-sectional community-based study. BMC Public Health. 2019 May 31 [cited 2023 Aug 9];19(1). Available from: /pmc/articles/PMC6544920/

4. Health Dossier 2021: Reflections on Key Health Indicators | National Health Systems Resource Centre. [cited 2023 Jul 19]. Available from: https://nhsrcindia.org/practice-areas/kmd/publications/health-dossier-2021

5. India - A-11 Appendix: District wise scheduled tribe population (Appendix), Jharkhand - 2011. [cited 2023 Jul 20]. Available from: https://censusindia.gov.in/nada/index.php/catalog/43019

6. Bango M, Ghosh S. Social and Regional Disparities in Utilization of Maternal and Child Healthcare Services in India: A Study of the Post-National Health Mission Period. Front Pediatr. 2022 Jun 14;10:895033.

7. Ali B, Chauhan S. Inequalities in the utilisation of maternal health Care in Rural India: Evidences from National Family Health Survey III & IV. BMC Public Health. 2020 Mar 20 [cited 2023 Jul 19];20(1):1–13. Available from: https://bmcpublichealth.biomedcentral.com/articles/10.1186/s12889-020-08480-4

8. Pannu Praveen, Gulati Nidhi, Yadav Neha. (PDF) “Accessibility of Reproductive Health Related Schemes for Pregnant and Lactating Rural Women in Uttar Pradesh: Some Reflections”. [cited 2023 Jul 19]. Available from: https://www.researchgate.net/publication/351578197_Accessibility_of_Reproductive_Health_Related_Schemes_for_Pregnant_and_Lactating_Rural_Women_in_Uttar_Pradesh_Some_Reflections

9. Chauhan BG, Jungari S. Factors Affecting the Utilization of Maternal and Child Health Care Services in Tribal Dominated Population States of India. https://doi.org/101177/0272684X20972857. 2020 Nov 17 [cited 2023 Jul 19];42(1):47–56. Available from: https://journals.sagepub.com/doi/abs/10.1177/0272684X20972857

10. Thongkong N, Van De Poel E, Roy SS, Rath S, Houweling TAJ. How equitable is the uptake of conditional cash transfers for maternity care in India? Evidence from the Janani Suraksha Yojana scheme in Odisha and Jharkhand. Int J Equity Health. 2017 Mar 10 [cited 2023 Jul 19];16(1):1–9. Available from: https://link.springer.com/articles/10.1186/s12939-017-0539-5

11. Chakraborty S. Talking about gender and sexual reproductive health rights of adolescents and youth in Jharkhand. Asian J Women Stud. 2019;25(3):468–81.

**FOR ISC evaluation only:**

**Quantify score of proposal**

**1-5 novelty of proposal:**

**1-5 relevance of proposal in national / international context:**

**1-5 content and quality of written proposal:**

**1-5 feasibility of the proposal:**

**1-5 experience of investigators:**

**1-5 infrastructure of departments/ institute to carry out the proposal**

**1-5 Quality of methods including statistics:**

**1-5 Potential for a larger project to emerge from this proposal**

**1-5 Multidisciplinary approach applied**

**1-5 Have the limitations/ risks/ challenges of the proposal been adequately addressed?**

**Total score of 50**

**Approved/ revision/ rejected:**

**Date:**

**Name of ISC member 1, signature and date:**

**Name of ISC member 2, signature and date:**

**Head, Research Cell, signature and date:**

**Dean, MTMC, signature and date**

Annexure 1:

Draft Questionnaires:

Note: Questionnaires is in the validation process.

Objective 1: To assess the knowledge, attitude, and practices of healthcare schemes among reproductive age group tribal women.

Section A: Socio Demographic profile

**1. Socio demographic profile:**

| **S.No.** | **Variable** | **Response** |
| --- | --- | --- |
| 1 | Age | - 15-29yrs - 29-39yrs - 39-49yrs |
| 2 | Residence | - Rural - Urban |
| 3 | Religion | - Hindu - Sarna Dharam - Christian - Muslim - Other |
| 4 | Ethnicity | - Tribal - Non tribal |
| 5 | Education | - Illiterate - Up to primary school - Secondary education - Above secondary |
| 6 | Occupation | - Self employed - Daily wage workers - Govt job - Private job - Other please specify |
| 8 | Marital status | - Married - Unmarried - Widowed - Divorcee - Separated |

Section B: Scheme related questions

|  |  | **Adolescent girls** | | | **Non-Pregnant Non Lactating** | | | | | **Pregnant and lactating** | | | | |
| --- | --- | --- | --- | --- | --- | --- | --- | --- | --- | --- | --- | --- | --- | --- |
| **S.No.** | **Questions** | **RKSK** | **Poshan Abhiyan** | **Anaemia Mukt Bharat** |  | **ASHA Scheme** | **Enhanced Compensatory Scheme** | **Anaemia Mukt Bharat** |  | **JSY** | **JSSK** | **PMMVY** | **PMSMA** | **Anaemia Mukt Bharat** |
|  |  |  |  |  |  |  |  |  |  |  |  |  |  |  |
|  | **Knowledge Questions** |  |  |  |  |  |  |  |  |  |  |  |  |  |
| 1 | Are you aware of the program? | Yes/No | Yes/No | Yes/No |  | Yes/No | Yes/No | Yes/No |  | Yes/No | Yes/No | Yes/No | Yes/No | Yes/No |
| 2 | Do you know the objectives and services provided under the program ? | Yes/No | Yes/No | Yes/No |  | Yes/No | Yes/No | Yes/No |  | Yes/No | Yes/No | Yes/No | Yes/No | Yes/No |
| 3 | If yes, please state them.. |  |  |  |  |  |  |  |  |  |  |  |  |  |
| 4 | Have you ever received any information or awareness campaign about the program in your locality, Aanganwadi centers, or through the hospital? | Yes/No | Yes/No | Yes/No |  | Yes/No | Yes/No | Yes/No |  | Yes/No | Yes/No | Yes/No | Yes/No | Yes/No |
| 5 | If yes, how did you receive the information | • Through ASHA • Through AWW • Family / friends • Posters • Television or radio • Community meetings • Others specify | • Through ASHA • Through AWW • Family / friends • Posters • Television or radio • Community meetings • Others specify | • Through ASHA • Through AWW • Family / friends • Posters • Television or radio • Community meetings • Others specify |  | • Through ASHA • Through AWW • Family / friends • Posters • Television or radio • Community meetings • Others specify | • Through ASHA • Through AWW • Family / friends • Posters • Television or radio • Community meetings • Others specify | • Through ASHA • Through AWW • Family / friends • Posters • Television or radio • Community meetings • Others specify |  | • Through ASHA • Through AWW • Family / friends • Posters • Television or radio • Community meetings • Others specify | • Through ASHA • Through AWW • Family / friends • Posters • Television or radio • Community meetings • Others specify | • Through ASHA • Through AWW • Family / friends • Posters • Television or radio • Community meetings • Others specify | • Through ASHA • Through AWW • Family / friends • Posters • Television or radio • Community meetings • Others specify | • Through ASHA • Through AWW • Family / friends • Posters • Television or radio • Community meetings • Others specify |
| 6 | Did you receive financial cash assistance? | Yes/No | Yes/No | Yes/No |  | Yes/No | Yes/No | Yes/No |  | Yes/No | Yes/No | Yes/No | Yes/No | Yes/No |
| 7 | If, Yes then how much money did you receive? |  |  |  |  |  |  |  |  |  |  |  |  |  |
| 8 | In whose account the money got credited? | Beneficiary account Husband account Parents account Inlaws account others.. | Beneficiary account Husband account Parents account Inlaws account others.. | Beneficiary account Husband account Parents account Inlaws account others.. |  | Beneficiary account Husband account Parents account Inlaws account others.. | Beneficiary account Husband account Parents account Inlaws account others.. | Beneficiary account Husband account Parents account Inlaws account others.. |  | Beneficiary account Husband account Parents account Inlaws account others.. | Beneficiary account Husband account Parents account Inlaws account others.. | Beneficiary account Husband account Parents account Inlaws account others.. | Beneficiary account Husband account Parents account Inlaws account others.. | Beneficiary account Husband account Parents account Inlaws account others.. |
| 9 | How the incentivised money was utlilized? Please specify |  |  |  |  |  |  |  |  |  |  |  |  |  |
|  | **Attitude** |  |  |  |  |  |  |  |  |  |  |  |  |  |
| 10 | Do you think the program is beneficial in improving healthcare services in your region? | • Strongly Agree • Somewhat Agree • Neutral • Somewhat disagree • Strongly disagree | • Strongly Agree • Somewhat Agree • Neutral • Somewhat disagree • Strongly disagree | • Strongly Agree • Somewhat Agree • Neutral • Somewhat disagree • Strongly disagree |  | • Strongly Agree • Somewhat Agree • Neutral • Somewhat disagree • Strongly disagree | • Strongly Agree • Somewhat Agree • Neutral • Somewhat disagree • Strongly disagree | • Strongly Agree • Somewhat Agree • Neutral • Somewhat disagree • Strongly disagree |  | • Strongly Agree • Somewhat Agree • Neutral • Somewhat disagree • Strongly disagree | • Strongly Agree • Somewhat Agree • Neutral • Somewhat disagree • Strongly disagree | • Strongly Agree • Somewhat Agree • Neutral • Somewhat disagree • Strongly disagree | • Strongly Agree • Somewhat Agree • Neutral • Somewhat disagree • Strongly disagree | • Strongly Agree • Somewhat Agree • Neutral • Somewhat disagree • Strongly disagree |
| 11 | In your opinion, how important is it for tribal women to receive the incentives through the program? | • Very Important • Somewhat important • Neutral • Not very important  • Not important at all | • Very Important • Somewhat important • Neutral • Not very important  • Not important at all | • Very Important • Somewhat important • Neutral • Not very important  • Not important at all |  | • Very Important • Somewhat important • Neutral • Not very important  • Not important at all | • Very Important • Somewhat important • Neutral • Not very important  • Not important at all | • Very Important • Somewhat important • Neutral • Not very important  • Not important at all |  | • Very Important • Somewhat important • Neutral • Not very important  • Not important at all | • Very Important • Somewhat important • Neutral • Not very important  • Not important at all | • Very Important • Somewhat important • Neutral • Not very important  • Not important at all | • Very Important • Somewhat important • Neutral • Not very important  • Not important at all | • Very Important • Somewhat important • Neutral • Not very important  • Not important at all |
| 12 | How would you rate the overall experience with the Program? | • Excellent • Good • Average • Poor | • Excellent • Good • Average • Poor | • Excellent • Good • Average • Poor |  | • Excellent • Good • Average • Poor | • Excellent • Good • Average • Poor | • Excellent • Good • Average • Poor |  | • Excellent • Good • Average • Poor | • Excellent • Good • Average • Poor | • Excellent • Good • Average • Poor | • Excellent • Good • Average • Poor | • Excellent • Good • Average • Poor |
| 13 | In your opinion what are the challenges and drawbacks of the scheme? |  |  |  |  |  |  |  |  |  |  |  |  |  |
|  | **Practices** |  |  |  |  |  |  |  |  |  |  |  |  |  |
| 14 | Would you recommend the scheme to other tribal women in your area? | • Yes • Unsure • No | • Yes • Unsure • No | • Yes • Unsure • No |  | • Yes • Unsure • No | • Yes • Unsure • No | • Yes • Unsure • No |  | • Yes • Unsure • No | • Yes • Unsure • No | • Yes • Unsure • No | • Yes • Unsure • No | • Yes • Unsure • No |
| 15 | Have you or your family members availed the benefits of these schemes? | Yes/No | Yes/No | Yes/No | Yes/No | Yes/No | Yes/No | Yes/No |  | Yes/No | Yes/No | Yes/No | Yes/No | Yes/No |
| 16 | If you answered "Yes" to the previous question, please provide details of your experience? |  |  |  |  |  |  |  |  |  |  |  |  |  |
| 17 | If you haven't availed benefits, what were the reasons for not utilizing the program? (Select all that apply) | Lack of awareness  Did not meet the eligibility criteria Preference for home delivery Lack of trust in the healthcare system Other (please specify) ______________ | Lack of awareness  Did not meet the eligibility criteria Preference for home delivery Lack of trust in the healthcare system Other (please specify) ______________ | Lack of awareness  Did not meet the eligibility criteria Preference for home delivery Lack of trust in the healthcare system Other (please specify) ______________ |  | Lack of awareness  Did not meet the eligibility criteria Preference for home delivery Lack of trust in the healthcare system Other (please specify) ______________ | Lack of awareness  Did not meet the eligibility criteria Preference for home delivery Lack of trust in the healthcare system Other (please specify) ______________ | Lack of awareness about JSY Did not meet the eligibility criteria Preference for home delivery Lack of trust in the healthcare system Other (please specify) ______________ |  | Lack of awareness  Did not meet the eligibility criteria Preference for home delivery Lack of trust in the healthcare system Other (please specify) ______________ | Lack of awareness  Did not meet the eligibility criteria Preference for home delivery Lack of trust in the healthcare system Other (please specify) ______________ | Lack of awareness  Did not meet the eligibility criteria Preference for home delivery Lack of trust in the healthcare system Other (please specify) ______________ | Lack of awareness  Did not meet the eligibility criteria Preference for home delivery Lack of trust in the healthcare system Other (please specify) ______________ | Lack of awareness  Did not meet the eligibility criteria Preference for home delivery Lack of trust in the healthcare system Other (please specify) ______________ |
| 18 | What factors do you think can encourage to utilize health schemes? |  |  |  |  |  |  |  |  |  |  |  |  |  |

Objective 2: To understand the challenges faced in provision of tribal reproductive age group women healthcare schemes from different Stakeholders perspective.

In-depth interview:

1. In your experience or based on your knowledge, what are the main challenges faced by eligible beneficiaries in utilizing the benefits provided by the healthcare schemes of tribal reproductive age group women?

a. Probe: barriers/obstacles, awareness, availability of schemes

2. What are the main challenges encountered in the distribution of the benefits of different schemes related to reproductive age group women?

a. Probe: Incentives, referral support, transportation in JSY, JSSK, PMMVY, PMMSA, RKSK, ASHA scheme, Compensatory scheme, Poshan Abhiyan, Anaemia Mukt Bharat.

b. Logistic, administrative hurdles.

3. In your experience or based on your knowledge, what are the operational challenges faced by healthcare providers or implementing agencies in delivering the services through the reproductive age group health coverage schemes?

a. Probe: challenges related to training of healthcare workers.

b. Probe: Challenges in ensuring availability of infrastructure, equipment and supplies.

4. We would like to know what are the challenges you faced during the registration processes of these health coverage schemes?

a. Probe: Challenges for document requirement, effectiveness of existing registration

5. We would like to know the challenges you encounter in monitoring and evaluating the implementation of these reproductive health coverage schemes?

a. Probe: difficulty in data collection, timely reporting

6. Could you tell us about the challenges associated with the disbursement of financial benefits under the JSY, JSSK, PMMVY, Enhanced Compensatory Schemes? Are there any delays or issues in ensuring timely payments to eligible beneficiaries?

a. Probe: Challenges related to financial management, budgeting, fund allocation, and utilization.

7. What challenges do you encounter in coordination and communicating with various stakeholder involved in these schemes, such as healthcare providers, implementing agencies, and government officials?

a. Probe: intersectoral coordination, communication, coordination, and cooperation (towards demand/supply, utilization)

8. Can you please through some lights on the challenges faced on maintenance of records of beneficiaries in these schemes?

a. Probe: audits (schedule period, team members involved), online records, register maintenance, specific suggestions.

9. What challenges do you observe in creating awareness about the reproductive health coverage schemes among eligible beneficiaries and key stakeholders?

a. Probe: difficulties in disseminating accurate/timely information, reach of awareness campaign, community support, community participation.

10. We would like to know about the challenges you observe in fostering effective collaboration and coordination among various stakeholders involved in these schemes, including government agencies, healthcare providers, and community organizations?

11. Can you please tell us the challenges associated with providing adequate training and capacity building for healthcare providers and staff involved in these schemes?

a. Probe: Was proper training given, too less or too hurried? Unplanned? Areas to improve in the training? In the present state what are the lacunae in training?

12. What challenges do you observe in the design of these reproductive age group women healthcare schemes that may affect its long-term sustainability?

a. Probe: changing healthcare needs, adequate funding, challenges encountered in institutional support, community engagement

13. We would like to know based on you experience or knowledge, what strategies or intervention would you recommend to address the challenges in the reproductive age group health coverage schemes?

|  |  |  | | |  | | | | |  | | | | | |
| --- | --- | --- | --- | --- | --- | --- | --- | --- | --- | --- | --- | --- | --- | --- | --- |
|  |  |  |  |  |  |  |  |  |  |  |  |  |  |  |  |
|  |  |  |  |  |  |  |  |  |  |  |  |  |  |  |  |
|  |  |  |  |  |  |  |  |  |  |  |  |  |  |  |  |
|  |  |  |  |  |  |  |  |  |  |  |  |  |  |  |  |
|  |  |  |  |  |  |  |  |  |  |  |  |  |  |  |  |
|  |  |  |  |  |  |  |  |  |  |  |  |  |  |  |  |
|  |  |  |  |  |  |  |  |  |  |  |  |  |  |  |  |
|  |  |  |  |  |  |  |  |  |  |  |  |  |  |  |  |
|  |  |  |  |  |  |  |  |  |  |  |  |  |  |  |  |
|  |  |  |  |  |  |  |  |  |  |  |  |  |  |  |  |
|  |  |  |  |  |  |  |  |  |  |  |  |  |  |  |  |
|  |  |  |  |  |  |  |  |  |  |  |  |  |  |  |  |
|  |  |  |  |  |  |  |  |  |  |  |  |  |  |  |  |
|  |  |  |  |  |  |  |  |  |  |  |  |  |  |  |  |
|  |  |  |  |  |  |  |  |  |  |  |  |  |  |  |  |
|  |  |  |  |  |  |  |  |  |  |  |  |  |  |  |  |
|  |  |  |  |  |  |  |  |  |  |  |  |  |  |  |  |
|  |  |  |  |  |  |  |  |  |  |  |  |  |  |  |  |
|  |  |  |  |  |  |  |  |  |  |  |  |  |  |  |  |
|  |  |  |  |  |  |  |  |  |  |  |  |  |  |  |  |
|  |  |  |  |  |  |  |  |  |  |  |  |  |  |  |  |
|  |  |  |  |  |  |  |  |  |  |  |  |  |  |  |  |
|  |  |  |  |  |  |  |  |  |  |  |  |  |  |  |  |
|  |  |  |  |  |  |  |  |  |  |  |  |  |  |  |  |
