# Supplementary material for: Development & validation of a health awareness booklet: “Reproductive health schemes for tribal women of Jharkhand” a study protocol
Source: PLoS One. 2025 May 5;20(5):e0322777. doi: 10.1371/journal.pone.0322777 (PMC12052126; doi:10.1371/journal.pone.0322777)
Supplement: Attachment — Submitted filename: Rebuttal letter.docx [file pone.0322777.s004.docx]

Dr. Jarina Begum

Professor, MBBS, MD, FAIMER

Email Id: jarina.begum@manipal.edu

23^rd^ December 2024

Editor-in-Chief

Plos One Journals

Subject: Rebuttal Letter for Manuscript ID “PONE-D-24-37230”

Dear George,

Thank you for providing the reviewers' comments on our manuscript titled: Development & Validation of a health awareness booklet: “Reproductive Health Schemes for tribal women of Jharkhand” a study protocol. We greatly appreciate the thorough evaluation and constructive feedback, which have helped us to enhance the quality of our work.

Below, we provide a detailed point-by-point response to the reviewers’ comments. Our revisions are highlighted in the revised manuscript for clarity.

Part 1 (Journal requirements):

1. Rechecked and updated the manuscript as per Plos One guidelines.

2. For data availability statement, there will be no data availability as this is a study protocol.

3. Supporting Information file without an institutional logo has been updated.

4. Copy of Table 1 has been updated in the text.

**Part 2 (Comments from the reviewer and updates) Review Comments to the Author**

Reviewer #1: A research study is being proposed which aims to explore the utilization of healthcare schemes among tribal reproductive-age groups in Jharkhand India with an ultimate goal of improving access to quality healthcare services. The research will be conducted in two phases. Phase one will to assess the knowledge, attitude, and practices of healthcare schemes as objective 1 and to understand facilitators and challenges in provision of tribal reproductive age group women healthcare schemes from different stakeholder perspectives. Phase two will develop and validate an awareness booklet.

Major revision:
Include a comprehensive statistical analysis plan detailing the analysis to be conducted for each phase of the study.

Ans: Comprehensive statistical analysis have been added in the main manuscript.

Minor revisions:
Identify the software that will be used for statistical analysis.

For quantitative software Jamovi will be used and for qualitative study software Atlas ti will be used.

Reviewer #2: 1. In the justification for the study the authors mention Uncontrolled fertility. However, the supporting data is only of contraceptive use. There is no mention of TFR or NRR. Without a high TFR the authors cannot comment on fertility.

Ans: Uncontrolled fertility has been reupdated in the main manuscript with low contractive usage.

2. Objectives - The objectives are listed as bullet points. But in the methodology section the authors mention obj 1,2 &3

Answer: Objectives have been updated as objective 1, 2, and 3.

3. There is no separate section with Description of the Study Setting for the reader to understand the context. We expect population information, tribes, languages spoken, livelihood, terrain, access to health care etc.

Ans: Separate section with description of the study setting has been added in the manuscript.

4. Study Design & Methodology-

4a. Sequential explanatory design uses qualitative data to explain quantitative data- This is partly done for the phase 1. A note on which aspects will be addressed using this design will help. Authors should clearly mention the quantitative component, the qualitative component and the booklet design and testing component.

Answer: For this, I would like to give my views as per my knowledge Sequential explanatory design uses quantitative data first, followed by qualitative data. Hence quantitative method we have mentioned in objective 1, and qualitative aspect in objective 2, based on the mixed result we will develop the booklet as per the need reported.

4b. What is the age group? 10-19, 15-49 is mentioned, but the revised protocol table only shows age starting from 15.

Ans: There are two groups, first adolescent girl group (10-19 years) and another is tribal women in the reproductive age group i.e. from 15 to 49 years.

Table has been updated with the correct age group.

4c. Religion - Why is Sarna not listed

In questionnaire Sarna Dharam has been included under religion and also updated in the main manuscript sampling section.

4d. Marital Status- Where do separated women get listed?

Separated women has been included in the questionnaire under marital status.

5. Questionnaire is Self administered. So the assumption is that all participants are literate. Then why is thumb impression needed?

Answer: A Self-administered questionnaire will be used, and in case of illiterate study participants, questionnaire form will be filled by the interviewer/ or the person who will be going to collect the data, on behalf of study participant. (this has been updated in the main manuscript) Data will be collected only after the consent given by the study participants. Hence in case of illiterate participant their thumb impression will be taken in the consent form.

6. Authors mention that the sampling is simple random sampling and stratified sampling. Which part is simple random sampling?

Out of two blocks, twelve villages will be chosen at random, six village from each block. From each village there will be stratified into two strata, tribal adolescent girls (n=32/village) and tribal women of reproductive age group. (n=30 per village).

7. What is the basis for a design effect of 1.5?

Ans: The design effect quantifies how much the variance of an estimate in a complex sampling design (such as stratified, clustered, or systematic sampling) exceeds that of a simple random sampling (SRS) design with the same sample size. Basis for Choosing a Design Effect of 1.5. Stratification by Two Groups (Tribal Women and Adolescent Girls) ensures representation of both groups, but variations in responses within each stratum (e.g., due to cultural, linguistic, or educational differences) may introduce design-related dependencies. Although stratification often improves precision, the overall sampling design may not achieve the same variance as a simple random sample, warranting a modest adjustment.

Also, the study population comprises tribal women and adolescent girls from a tribal community. These groups may have unique socio-cultural factors, such as resistance to participation, communication barriers, variations in awareness or understanding of the study. These factors increase the likelihood of non-participation or clustering effects, justifying an elevated design effect. One of the major problem will be non-response or refusal to participate in studies is common in marginalized or hard-to-reach populations, leading to an effective reduction in sample size and potential clustering of respondents within more cooperative subgroups. A design effect accounts for the loss of randomness in sampling due to these patterns. Hence, as per the Biostatistician, suggestion a design effect of 1.5 reflects a reasonable compromise between underestimating and overestimating the effect of design-related variability.

8. A simple explanation for using Phenomenology approach must be added.

Ans: Updated in the main manuscript under method section. (Phenomenological approach, through this study participant can express there experienced field perspectives related to facilitators and challenges faced).

9. Working with the marginalised community - There is no mention of how the researchers plan to observe Cultural Sensitivity (I see that all authors are non-tribals). It is mentioned that permission from MO will be taken for the field research. Are the authors sure about how to approach to the tribal communities? An approach mentioned in the paper will only give screwed results because the approach is from health sector.

Answer: Yes, permission from State tribal welfare commissioner, District Surgeon, and District collectorate has been taken. To approach the tribal community we have taken the permissions from gatekeeper also these gatekeeper include Panchayat head (Mukhiya), and Village head (Gram Pradhan). With all the approved letters we will be going to the local health care providers such as ASHA and AWW, through them we will approach the community.

10. Implementation- The paper mentions Implementation. It is confusing. Implementation of what? Is the phase 2 about developing a booklet and testing it, or something else?

Ans: Phase 2 is about developing a booklet and testing it. After testing it can be give to the government health department for the implementation of the developed booklet.

Is it content and media development for a booklet or strategy, content, method and media development and trial?

Ans: The main focus of the booklet will be on the content with focus on pictorial form. This can be visualise and understand easily by the tribal communities. Hence it is a content and media development basically.

11. How many languages is the booklet going to be in? Which are the main languages spoken?

Ans: Booklet will be in two language, i.e. in English and in Hindi language. And the audio clips of the case studies in the booklet will be in tribal language i.e. In Santhali Language. (As in Jharkhand Santhal tribes are on the majority. But other language such as Sabar, Ho, Mundari can be use after the data collection as per the need.

12. Who will use the booklet and how will it be tested?

Ans: The developed booklet and the audio clips of the case studies in the booklet will be given to the healthcare providers such as ASHA, Aanganwadi workers, and ANM. Tribal women or adolescent girls whoever want the information about the scheme can contact their near by healthcare providers and can get the information.

It will be tested in two ways:

1. Review from the experts, such as Medical Officers, Professors from the Public health domain, stakeholders from the ministerial level, and the grassroot health care providers.

2. Piloting of the booklet inform of awareness sessions to the study participant i.e. tribal women and adolescent girls, will be done in 10 percent of the total mentioned population.

We hope that the revisions we have made satisfactorily address all concerns raised by the reviewers. Additionally, we are happy to make further changes if needed. Thank you again for the opportunity to revise and resubmit our manuscript. Please do not hesitate to contact us if additional clarification or information is required.

Regards,

Dr. Jarina Begum (Corresponding author)

Professor

Department of Community medicine

Manipal Tata Medical College, Jamshedpur

Email: jarina.begum@manipal.edu

&

Dr. Rohit Raj (First author)

Ph.D. Research Scholar

Manipal Tata Medical College, Jamshedpur

Email: rohit1.mtmcjsr2022@learner.manipal.edu
