# Supplementary material for: Development & validation of a health awareness booklet: “Reproductive health schemes for tribal women of Jharkhand” a study protocol
Source: PLoS One. 2025 May 5;20(5):e0322777. doi: 10.1371/journal.pone.0322777 (PMC12052126; doi:10.1371/journal.pone.0322777)
Supplement: Attachment — Submitted filename: Rebuttal letter (1).docx [file pone.0322777.s005.docx]

Dr. Jarina Begum

Professor, MBBS, MD, FAIMER

Email Id: jarina.begum@manipal.edu

18^th^ March 2025

Editor-in-Chief

Plos One Journals

Subject: Rebuttal Letter for Manuscript ID “PONE-D-24-37230”

Dear George,

Thank you for providing the reviewers' comments on our manuscript titled: Development & Validation of a health awareness booklet: “Reproductive Health Schemes for tribal women of Jharkhand” a study protocol. We greatly appreciate the thorough evaluation and constructive feedback, which have helped us to enhance the quality of our work.

Below, we provide a detailed point-by-point response to the reviewers’ comments. Our revisions are highlighted in the revised manuscript for clarity.

References have been corrected and refined as per the suggestion.

We hope that the revisions we have made satisfactorily address the concerns raised by the reviewers. Additionally, we are happy to make further changes if needed. Thank you again for the opportunity to revise and resubmit our manuscript. Please do not hesitate to contact us if additional clarification or information is required.

I kindly request for full waive off if the protocol gets select. As I (Dr Rohit Raj) am a full time PhD. Scholar and still no funding is available yet.

Kind Regards,

Dr. Jarina Begum (Corresponding author)

Professor

Department of Community medicine

Manipal Tata Medical College, Jamshedpur

Email: jarina.begum@manipal.edu

&

Dr. Rohit Raj (First author)

Ph.D. Research Scholar

Manipal Tata Medical College, Jamshedpur

Email: rohit1.mtmcjsr2022@learner.manipal.edu
